# Counterfactual thinking in moral judgment: an experimental study

**DOI:** 10.3389/fpsyg.2014.00451

**Published:** 2014-05-20

**Authors:** Simone Migliore, Giuseppe Curcio, Francesco Mancini, Stefano F. Cappa

**Affiliations:** ^1^Integrated Center of Research, University Campus Bio Medico di RomaRome, Italy; ^2^School of Cognitive Psychotherapy (SPC)Rome, Italy; ^3^Department of Life, Health and Environmental Sciences, University of L' AquilaL' Aquila, Italy; ^4^Division of Neuroscience, San Raffaele Scientific InstituteMilan, Italy; ^5^Center for Neurocognition and Theoretical Syntax (NeTS), IUSS Istituto Universitario di Studi SuperioriPavia, Italy

**Keywords:** moral dilemma, decision making, gender, aging, utilitaristic reasoning

## Abstract

Counterfactual thinking is thinking about a past that did not happen. This is often the case in “if only…” situations, where we wish something had or had not happened. To make a choice in a moral decision-making situation is particularly hard and, therefore, may be often associated with the imagination of a different outcome. The main aim of the present study is to investigate counterfactual thinking in the context of moral reasoning. We used a modified version of Greene's moral dilemmas test, studying both the time needed to provide a counterfactual in the first and third person and the type of given response (in context-out of context) in a sample of 90 healthy subjects. We found a longer response time for personal vs. impersonal moral dilemmas. This effect was enhanced in the first person perspective, while in the elderly there was an overall slowing of response time. Out of context/omissive responses were more frequent in the case of personal moral dilemmas presented in the first person version, with females showing a marked increase in this kind of response. These findings suggest that gender and perspective have a critical role in counterfactual thinking in the context of moral reasoning, and may have implications for the understanding of gender-related inclinations as well as differences in moral judgment.

## Introduction

While people ponder about the choices they have taken, the thought “if only…,” often comes to their mind. We frequently construct counterfactual alternatives about what might have been. Such counterfactual thinking is pervasive in everyday life, and it has been examined by philosophers and psychologists (Stalnaker, 1968; Kahneman et al., 1982; Roese, 1997; Byrne and Tasso, 1999; Roese and Olson, 2014). Counterfactual thinking helps people to learn from experience and can influence different cognitive activities such as deduction, decision making, probability calculation and problem solving (Byrne, 2002; Coricelli et al., 2007; Epstude and Roese, 2008). Moreover, counterfactual thinking is associated with complex emotions, such as guilt, regret or blame (Camille et al., 2004; Young and Koenigs, 2007). Counterfactual reasoning may play an important role in moral judgment, in fact investigating the link between counterfactual thinking and moral judgment could increase our understanding of how people tend to condemn crimes, and thus be relevant for criminal and forensic psychology (i.e., how judges, courts and popular jury in general tend to condemn or absolve incriminated people). The literature addressing this issue is very limited. Research on counterfactual thinking (i.e., the construction of mental alternatives to reality) has established that some mental alternatives to the negative outcome of a scenario are more available than others (Byrne, 2005; Mandel et al., 2005; Roese, 2005). For example, Girotto et al. (1991) showed that events under the control of the protagonist, such as his/her decisions, are more amenable to mental change than events the protagonist cannot control. Moreover, the individual's role - in particular, whether the individual is an “actor” (i.e., directly involved) or a “reader” (i.e., an observer)- can strongly affect counterfactual thinking, and this could be due to different motivational goals between actors and readers (Elster, 1999; Gilbert et al., 2004). According to Girotto et al. (2007) there is another reason to posit differences between actors' and readers' counterfactual thoughts, i.e., the differential availability of information. Unlike readers, who only know the final outcome, the actors can easily retrieve from memory many elements of the problem-solving phase of the event. These elements concern salient parts of the actor's experience, and will determine the construction of an alternative choice. Nadelhoffer and Feltz (2008) conducted a behavioral study using a version of “trolley problem” (Thomson, 1976). The dilemma was presented either in the first person (actor) or in the third person (observer). In the actor condition, 65% of the participants found the action (throwing the switch) to be permissible, whereas in the observer condition 90% of the participants found the action as morally acceptable. Furthermore, Avram et al. (2014) demonstrated that the first or third person perspective in moral cognition involves distinct neural processes. While common activation was present in the anterior medial prefrontal cortex, the third person perspective elicited additional activations in hippocampus and visual cortex. The findings of these studies suggest that, in moral judgment, different psychological processes and brain networks are involved by the two perspectives.

In the same vein, different brain areas are also involved in personal and impersonal moral dilemmas. In fact, Greene et al. (2001) investigated the brain networks implicated in personal and impersonal moral dilemmas. They considered a moral violation to be personal if it meets the following three criteria: it must be likely to cause serious bodily harm; this harm must befall a particular person or set of persons; the harm must not result from the deflection of an existing threat onto a different party. Dilemmas that fail to meet these three criteria were classified as impersonal. Using fMRI, they reported a significant increase of cerebral activity when the subjects considered personal moral dilemmas, particularly in brain networks associated with emotion and social cognition (i.e., medial prefrontal cortex, posterior cingulate, superior temporal sulcus/temporo-parietal junction). In the case of impersonal moral dilemmas, increase in brain activity involved only neural networks subserving working memory and cognitive processes (i.e., dorsolateral prefrontal cortex, right dorsolateral prefrontal cortex (DLPFC), bilateral inferior parietal lobule). Moreover, Greene et al. (2004) showed that when subjects provide utilitarian responses in personal moral dilemma, in which utilitarian consequences of choices (i.e., maximize the gain, as to save 5 people instead 1) are in conflict with deontological rules (avoiding to harm others), the right DLPFC (involved in planning and reasoning) is activated, highlighting the influence of cognitive processes on utilitarian choices. While intuitively appealing, the personal/impersonal distinction has been criticized on several grounds. It is poorly generalizable to other classes of moral dilemmas (McGuire et al., 2009) and it cannot explain the variability found in response to the trolley problems (Mikhail, 2007). In addition, personal moral dilemmas use more emotive language and references to family members or friends than impersonal dilemmas, there is no control for cognitive processing requirements across conditions, and questioning whether the chosen actions were appropriate may be ambiguous (Schaich Borg et al., 2006).

The main aim of the present study is to investigate counterfactual thinking under the condition of moral reasoning, two phenomena intimately interrelated. Few studies investigated the link and mutual influence between these two processes; moreover, we know little about the role and influence of perspective, age and gender in this specific field.

As highlighted by Branscombe et al. (1996), moral judgment is strongly influenced by participants' focus in their counterfactual thinking. They showed that focusing on the assaillant's or the victim's behavior changes significantly their point of view about condemnation of the crime. Moreover, looking at the influence of moral judgment on counterfactual thinking, it was found that people tended to produce more counterfactual thinking for “immoral” than for neutral dilemmas (N'gbala and Branscombe, 1997). In fact, when individuals have to cope with a moral dilemma, they tend to focus on the agent who performed the action and are much more inclined to look at alternative actions that the agent could have performed. Moreover, counterfactuals reveal contradictions in our belief systems and highlight double standards in our moral judgments, being also necessary to evaluate the moral benefits of real world outcomes (Lebow, 2007). Finally, as shown by several studies, counterfactuals are useful in planning sub-goals and learning from experiences, thus acting as a guide in moral judgment (Epstude and Roese, 2008).

Such relationship between counterfactual thinking and moral judgment can be modulated by some individual differences such as age and gender. As shown previously, moral judgment presents strong gender-related differences, both at behavioral and neurobiological levels (Harenski et al., 2008; Fumagalli et al., 2010b), and such gender-related differences reflect two different theoretical approaches (Kohlberg, 1964; Gilligan, 1982). A recent study of Fumagalli et al. (2010a) found that responses to moral personal dilemmas differ specifically and selectively in the two genders, showing that men tend to provide more utilitarian answers than women, and that this peculiarity is independent of cultural factors such as education and religion. Regarding age, there is evidence of a developmental progression from childhood to adulthood in countefactuals production (Rafetseder et al., 2010), and it has been show that the ability to apply counterfactual reasoning is not fully developed in all children before 12 years of age (Rafetseder et al., 2013). Although such research suggests that age and gender may influence moral judgment, it remains unclear how much of this effect could be attributed to differences in counterfactual reasoning.

Finally, counterfactual thinking is the ability to create an imagined world as close as possible to the actual one, and this needs a change in some features of the actual world (Woodward, 2011). In this regard, Girotto et al. (2007) stated that a possible difference between different roles is related to the way used to change the actual world. Actors (who are directly involved in the situation) prefer large modifications, introducing elements not present in the real experience. When and under which circumstances people change these antecedent features and when they do not can be investigated by evaluating the presence or not of out of context answers, i.e., those including elements not present in the real world.

In the present study we evaluated the time needed to offer a counterfactual thought in different conditions (Non Moral, NM; Moral Impersonal, MI; Moral Personal, MP) and in the case of “first person” (actor) or “third person” (reader) perspective. We assessed the impact of individual differences in age and gender on the time required for response. Moreover, we also assessed the quality of answer (in context vs. out of context) and the possible effects induced by individual differences on this aspect of the response.

## Materials and methods

### Participants

Ninety healthy subjects (45 females) participated in the study. Their mean age was 46.83 ± 13.81 (range 20–70 years). Any neurological or psychiatric history and medication or drug intake was ruled out by means of a questionnaire and a clinical interview. All participants were Italian, and their mean education was at least 8 years (14.56 ± 3.27, range 8–18 years). Each participant was assigned to one of three age groups (Young, Adult, Elderly): information about the three subgroups is reported in Table 1.

**Table 1:** **Demographic information of the participants**.

	**Age**	**Education**
Overall sample (90 participants; 45 females)	46.83 ± 13.81	14.55 ± 3.27
Males	47.02 ± 14.16	14.36 ± 3.57
Females	46.64 ± 13.61	14.56 ± 2.97
Group 1: Young (25 < *x* < 39 years) (30 participants; 15 females)	30.23 ± 4.38	15.07 ± 2.68
Males	30.6 ± 5.41	15.07 ± 3.03
Females	29.87 ± 3.20	15.07 ± 2.37
Group 2: Adult (40 < *x* < 55 years) (30 participants; 15 females)	48.17 ± 4.38	13.43 ± 3.5
Males	47.4 ± 3.96	13.33 ± 3.99
Females	48.93 ± 4.77	13.53 ± 3.04
Group 3: Elderly (56 < *x* < 70 years) (30 participants; 15 females)	62.1 ± 4.36	15.17 ± 3.39
Males	63.07 ± 4.50	14.67 ± 3.02
Females	61.13 ± 4.16	15.67 ± 3.20

All participants signed an informed consent before participating in the study, and the study protocol was conducted in accordance with the Declaration of Helsinki.

### Methods

The main aim of the present study is to investigate the influences of selected variables on counterfactual thinking under the condition of moral judgment. To this extent, we started from the validated moral dilemmas task proposed by Greene et al. (2001), slightly modifying them to fit the purposes of the present experiment. In the original manuscript, this task includes a total of 64 dilemmas: 20 non moral (NM) dilemmas, where there is no emotional involvement in the generation of a counterfactual of the story; 19 impersonal (MI) dilemmas, where the protagonist does not cause or induce damages to others with his actions, but the emotional involvement is higher than in the NM ones; 25 personal moral (MP) dilemmas where the protagonist behaves in ways that can induce harm to others, but with good and positive purposes (i.e., to save someone).

First, we paired the number of dilemmas proposed to the participants (20 for each condition), trying to maintain the same structure of those of Greene et al. (2001). More specifically, we maintained the 20 NM dilemmas as in Greene et al, work, and we added one MI dilemma and deleted 5 of the MP dilemmas used in the Greene et al. study. The only MI dilemma included in this study was developed by our own and tested in a previous pilot study (unpublished results). Regarding MP dilemmas, we decided to exclude those that in the original study were present in two modified versions, in order to avoid an excessive similarity between different dilemmas.

Since Greene's dilemmas, in their original version, were not suitable to generate counterfactual thinking, we modified their final part in order to present to the participant a given situation. Differently from Greene and colleagues' paper, where a judgment of appropriateness was requested (i.e., *Is it appropriate for you to push the stranger on to the tracks in order to save the five workmen?*), here each participant was asked to cope with a situation in which a decision has already been taken, with the related consequences, and to propose a counterfactual thought, i.e., “What could they have done in order to avoid the given situation?” (i.e., Given situation: *Five people die.* Request for a counterfactual thought: *What could you have done to avoid this?)*. To stress conterfactual thinking, we proposed the worst scenario as the predefined situation.

Finally, in each group of 20 dilemmas 10 were created with a first person involvement/perspective (actor) and 10 with a third person involvement/perspective (reader). The whole set of dilemmas is reported in the Supplementary Material.

The time required to offer an answer was recorded, and the response was categorized as “in context” (i.e., “*I would have pushed the very large stranger off the bridge”*) or “out of context” (i.e., “*I would have asked the very large stranger to help me to lift a big stone”*). We considered the counterfactual answer to be “out of context” if it meets two criteria: first, the answer include elements not considered in scenario; second, these elements are used to avoid the given situation. Answers that fail to meet these two criteria were classified as “in context.”

Coherently with Greene et al.'s results (Greene et al., 2004) we hypothesized an increase in reaction times (RT) along the different types of dilemmas (NM < MI < MP), as a reflection of the conflict between utilitarian gain and deontological rules, the latter having a strong emotional value. Moreover, according to Girotto and colleagues' results (Girotto et al., 2007) we hypothesized an increase of “out of context” answers in case of “first person” (actor) situation with respect to the involvement as third person (reader). Finally, we assessed the effect of gender and age.

### Procedure

**Figure 1:**
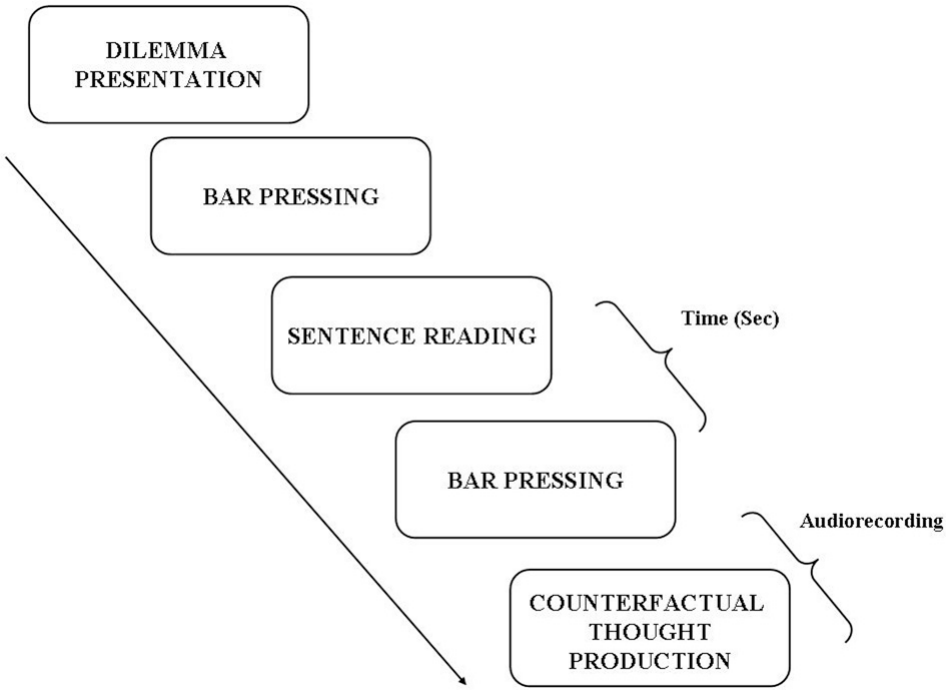
**Experimental protocol**.

Participants were tested in a sound-proof, temperature controlled environment. Dilemmas were presented in a randomized order by means of a dedicated software (Superlab 4.0 for Windows) that allows recording RTs needed to generate a counterfactual thought. All experimental sessions were also audio-recorded in order to collect the response given by the participants. Each subject was positioned in front of a computer screen at a distance of 50–60 cm. Before the start of recording session, instructions were presented on the screen: in case of need the participant could request more information to the experimenter. Then, the session started with the first dilemma. Participants were invited to read it carefully (without time limit) and only when it was completely clear he/she pressed a button on the keyboard and read the sentence related to the given situation. Starting from this screen presentation the time was recorded until the participant pressed a second time the button on the keyboard; immediately after he/she was asked to orally generate a counterfactual thought, that was audio recorded (see Figure 1). The provided counterfactual thought was then categorized as “in context” Vs “out of context” (see above).

### Statistical analyses

Response time and type of answer were entered in a mixed ANOVA: Gender (M, F) × Age (Young, Adults, Elderly) × Condition (NM, MI, MP dilemmas) × Perspective (1st person, 3rd person). Because of the high number of comparisons, Bonferroni correction was applied and significance level set at *p* < 0.02 for RTs and to *p* < 0.01 for type of answer provided.

## Results

ANOVA on response times showed a statistically significant main effect for Age [*F*_(2, 84)_ = 7.89; *p* = 0.0007], indicating that time needed for responding was higher in the Elderly (*M* = 16.89 ± 0.83) with respect to the other groups (Adults: *M* = 12.20 ± 0.74; Young *M* = 12.58 ± 0.91). *Post-hoc* comparisons (Scheffè' test) indicated that Elderly were significantly different from Adult (*p* = 0.002) and Young (*p* = 0.007), while Adult and Young participants did not differ in the time needed to provide a counterfactual thought.

Another significant main effect was related to Condition [*F*_(2, 168)_ = 75.61; *p* < 0.000001]; in this case an increase in response time was observed from NM (*M* = 10.59 ± 0.51), to MI (*M* = 14.41 ± 0.50) and to MP (*M* = 16.17 ± 0.61): MP dilemmas required much more time to express a counterfactual. *Post-hoc* comparisons showed that NM dilemmas were significantly different (*p* < 0.000001) from both MI and MP, and that MI and MP also differed (*p* = 0.03).

**Figure 2:**
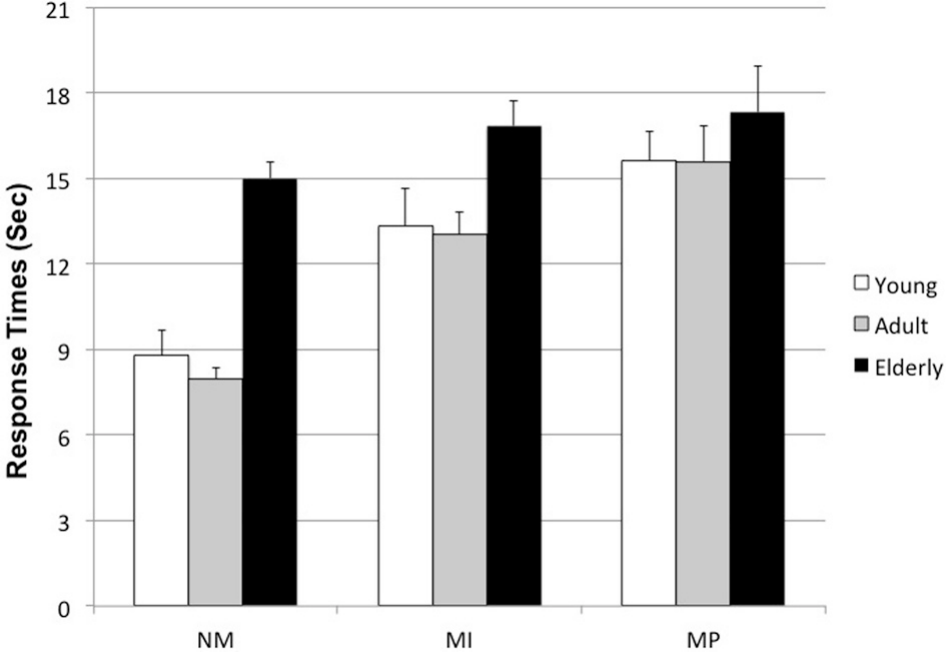
**Condition × Age interaction on Response time**. (NM, Non Moral; MI, Moral Impersonal; MP, Moral personal. Young: 25 < *x* < 39 years; Adult: 40 < *x* < 55 years; Elderly: 56 < *x* < 70 years).

A Condition × Age interaction was also statistically significant [*F*_(4, 168)_ = 6.44; *p* < 0.00008], indicating that Elderly participants were generally slower than others, and this was true independent of the type of dilemma taken into consideration (see Figure 2; *post-hoc*: 0.01 < p < 0.00006).

**Figure 3:**
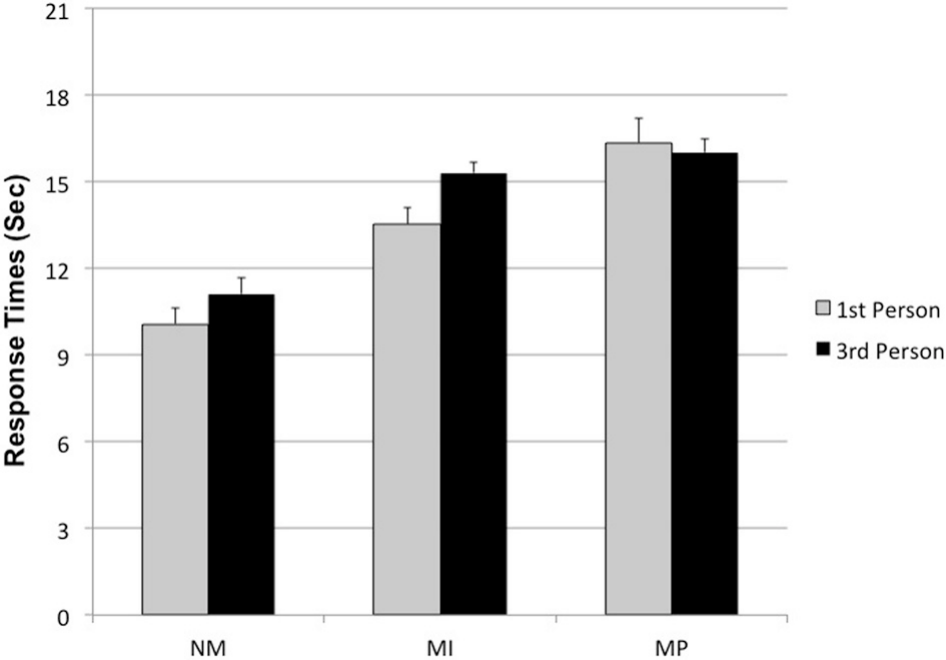
**Condition × Perspective interaction on Response time**. (NM, Non Moral; MI, Moral Impersonal; MP, Moral personal).

The interaction Condition × Perspective also reached statistical significance [*F*_(2, 168)_ = 9.32; *p* = 0.0001], indicating that MP dilemmas need a longer response time with respect to both NM and MI ones, and that this effect was boosted in the 1st person with respect to 3rd person perspective (see Figure 3).

No other statistically significant effect was found.

The ANOVA on type of answer (i.e., “out of context” answers) showed a significant main effect for Condition [*F*_(2, 168)_ = 103.38; *p* < 0.000001]. More specifically, a progressive increase in out of context answers was observed from NM (*M* = 0.01 ± 0.007), to MI (*M* = 0.94 ± 0.08) up to MP dilemmas (*M* = 1.44 ± 0.11). *Post-hoc* comparisons, in fact, showed that NM dilemmas were different by both MI and MP ones (*p* < 0.000001), as well as MI from MP dilemmas (*p* = 0.002).

A significant main effect for Gender was also shown [*F*_(1, 84)_ = 12.52; *p* = 0.0007], indicating that females produced more out of context answers (*M* = 0.98 ± 0.07) than males (*M* = 0.61 ± 0.08).

**Figure 4:**
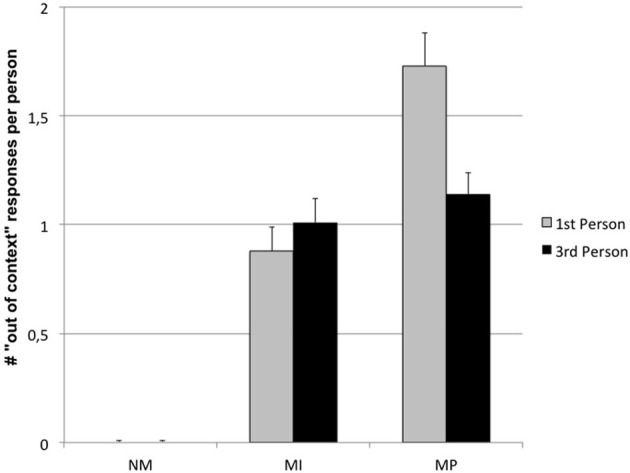
**Condition × Perspective interaction on “Out of context” responses**. (NM, Non Moral; MI, Moral Impersonal; MP, Moral personal).

A significant interaction Condition × Perspective was also found [*F*_(2, 168)_ = 10.98; *p* = 0.00003]. MP dilemmas were associated with an higher number of out of context counterfactual thoughts than other dilemmas, and this effect was amplified in the 1st person (actor) perspective, as shown in Figure 4 (*post-hoc p* = 0.0001).

**Figure 5:**
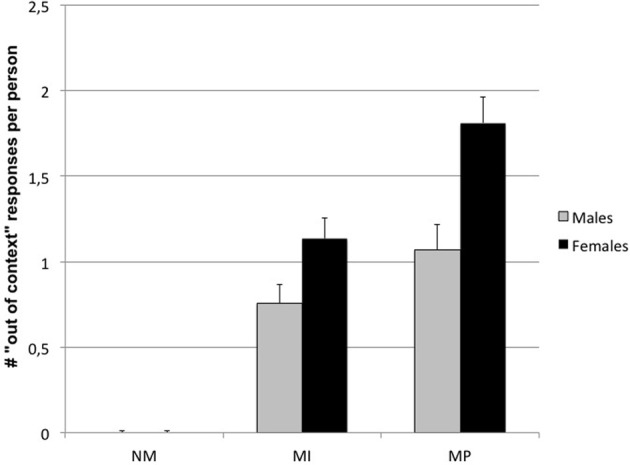
**Condition × Gender interaction on “Out of context” responses**. (NM, Non Moral; MI, Moral Impersonal; MP, Moral personal).

Finally, a significant Condition × Gender interaction has been observed [*F*_(2, 168)_ = 7.22; *p* = 0.0009], indicating that females produced an higher number of out of context answers than males, in particular for MI and MP dilemmas (0.0002 < p < 0.02) (see Figure 5).

## Discussion

This study aimed at investigating the impact of selected variables on counterfactual thinking in moral reasoning. For this purpose, we used a modified version of Greene's moral dilemmas test, assessing both the time needed to provide a counterfactual and the type of given response. Our results indicate an increase in time needed to offer a counterfactual when individuals have to cope with a MP dilemma. Such a slowing effect is amplified when individuals have to deal with moral dilemmas presented in the first person (i.e., actor). Moreover, looking at the type of answer, an higher number of “out of context” responses was given when individuals had to respond to moral personal dilemmas presented in first person version. In the latter case, gender was a relevant feature, since females showed a marked increase in this kind of response.

The progressive increase in time of response from NM up to MP dilemmas is in agreement with previous literature (Greene et al., 2001). In fact, counterfactual thinking in the context of moral reasoning depends both on cognitive and emotional processes. Following Greene et al. (2001), an action is morally right if it produces the highest utility of any available alternative action. The dual-process theory (Greene et al., 2001; Greene, 2009; McGuire et al., 2009) hypothesizes the presence of both an affective system activated by moral personal judgments and of a cognitive system preferentially activated by impersonal conflicts. Our finding may reflect a conflict between deontological rules and cognitive control of problem solving. Counterfactual thinking takes a longer time in the moral vs. non-moral conditions because the involved emotional status is much stronger and can intensify this conflict. Counterfactual thinking plays a largely beneficial function for behavior regulation (Johnson and Sherman, 1990), leading to performance improvement (Epstude and Roese, 2008). Nevertheless, in a context of moral reasoning, conflict may explain the increase in response time revealed in moral conditions.

Our study also showed a significant age-related difference. The significant increase of response times observed in elderly subjects may indicate a cognitive slowing (Deary et al., 2010; Eckert, 2011). This effect is significant both in contexts of moral and non-moral judgment supporting the idea that it could be an specific consequence of normal aging.

The present study also showed some differences with respect to the type of response provided, with an increased number of “out of context” responses in the moral dilemmas. This finding reflects the tendency to use elements that are not directly present in scenario, when a major conflict is present. This strategy is aimed to have a broader choice for decision-making. Furthermore, this tendency is greater in case of personal moral dilemmas when presented in first person perspective. Our data integrate those obtained in some previous studies (Elster, 1999; Gilbert et al., 2004; Girotto et al., 2007), in which the role played by the subject (actor or reader of the story) influenced considerations and choices in counterfactual thinking. For example, Elster (1999) argued that in the case of traumatic events, actors will mentally modify external events rather than their own actions, because they are motivated to avoid blame for their needs. Gilbert et al. (2004) asked individuals who actually missed their train by 1 min to reason counterfactually. These actors constructed external counterfactuals more often than passengers who merely had to imagine having missed their train. The authors' interpretation is that actors and readers had different motivational goals. There is, however, another reason to suppose differences between actors' and readers' counterfactual thoughts: the differential availability of salient information (Girotto et al., 2007). Here the authors conclude that actors and readers produce different counterfactuals even because they rely on different information, independently by motivations.

Another relevant aspect of moral dilemmas processing is related to gender differences. We found that women provided more out of context answers, “getting around” the conflict. More specifically, women implicated as first person in moral dilemmas (MI and MP) provides a greater number of out of context responses than men. This finding supports the idea that women are less inclined to make utilitarian choices, trying to avoid putting others at risk of danger or harm (Gilligan, 1982). Following this point of view, Gilligan hypothesized that women could be mainly driven by emotions, empathy and care for others, following the so-called ethics of care, while men could tend to solve moral dilemmas following law and order rules, according to an “ethics of justice.” It has also been suggested that these gender-related differences could be connected to differences in empathic ability (Baron-Cohen and Wheelwright, 2004; Eisenberg, 2005), which make females more resistant to decisions that entail directly inflicting physical or moral pain to other individuals, despite their utilitarian value. The present findings are in line with data proposed by Fumagalli et al. (2010a) suggesting that different cognitive–emotional processes, possibly reflecting differences in the underlying neural mechanisms, are involved in evaluating MP dilemmas in men and women. A functional neuroimaging study (Harenski et al., 2008) investigated the neural correlates of moral sensitivity in females and males, and found increased posterior cingulate cortex and anterior insula activation in females, and an enhanced inferior parietal cortex activation in males. A remarkable gender-related difference has been found also in specific neurotransmitter system, in particular in the frontal lobe neurotransmitters related to behavior (Kaasinen et al., 2001; Riccardi et al., 2005). In addition, hormones greatly influence behavior and their receptor distribution differs between sexes in the brain structures involved in cognition (Kimura, 2002; Cahill, 2006).A further demonstration that gender and its neuroanatomical bases is a key feature in moral decision making, has been recently proposed by Fumagalli et al. (2010b). By using transcranial direct current stimulation (tDCS) applied to the ventral prefrontal cortex (VPC), the authors found that the stimulation can interfere with utilitarian decisions, influencing the evaluation of the advantages and disadvantages of each option. While this effect is seen in both sexes, it is stronger in females. The authors conclude that gender-related tDCS-induced changes suggest that VPC differentially controls utilitarian reasoning in females and in males.

The present study has several limitations. One is related to the limited sample size. A higher number of participants could have given the possibility to show other effects or interactions between factors under investigation. The choice to apply a multiple comparison adjustment, however, can be considered as an adequate protection from a type I error. Another possible limitation is related to the choice of stressing counterfactual thinking, by always proposing to the participants the worst scenario as the predefined situation. Unfortunately, it was not possible to present both the best and worst scenarios, in order to avoid a desensitization of participants to the moral questions. Presenting only the best case (only 1 death instead of 5, as in the trolley dilemma) could have induced in the participant a kind of “satisfaction” to be in the best possible situation.

In conclusion, our study suggests that age, gender and perspective have a critical role in moral judgment and associated counterfactual thinking. This finding may have implications for the understanding of gender-related inclinations (i.e., specific job choice, leadership and power management) as well as differences in moral behavior (i.e., criminal behaviors). Future studies need to be specifically designed to investigate the neuroanatomical and neurophysiological differences of counterfactual thoughts in moral decision-making, as well as the emotional consequences, such as feelings of guilt, related to choices made in specific conditions.

### Conflict of interest statement

The authors declare that the research was conducted in the absence of any commercial or financial relationships that could be construed as a potential conflict of interest.

## References

[B1] Avram M. Hennig-Fast K. Bao Y. Pöppel E. Reiser M. Blautzik J. (2014). Neural correlates of moral judgments in first- and third-person perspectives: implications for neuroethics and beyond. BMC Neurosci. 1:39 10.1186/1471-2202-15-39 24742205 PMC3991864

[B2] Baron-Cohen S. Wheelwright S. (2004). The empathy quotient: an investigation of adults with asperger syndrome or high functioning autism, and normal sex differences. J. Autism Dev. Disord. 34, 163–175 10.1023/B:JADD.0000022607.19833.00 15162935

[B3] Branscombe N. Owen S. Garstka T. Coleman J. (1996). Rape and accident counterfactuals: who might have done otherwise and would it have changed the outcome? J. Appl. Soc. Psychol. 26, 1042–1067 10.1111/j.1559-1816.1996.tb01124.x

[B4] Byrne R. M. Tasso A. (1999). Deductive reasoning with factual, possible, and counterfactual conditionals. Mem. Cognit. 27, 726–740 10.3758/BF03211565 10479830

[B5] Byrne R. M. J. (2002). Mental models and counterfactual thoughts about what might have been. Trends Cogn. Sci. 6, 426–431 10.1016/S1364-6613(02)01974-5 12413576

[B6] Byrne R. M. J. (2005). The Rational Imagination: How People Create Alternatives to Reality. Cambridge, MA: The MIT Press 10.1017/S0140525X07002579 18321404

[B7] Cahill L. (2006). Why sex matters for neuroscience. Nat. Rev. Neurosci. 7, 477–484 10.1038/nrn1909 16688123

[B8] Camille N. Coricelli G. Sallet J. Pradat-Diehl P. Duhamel J. R. Sirigu A. (2004). The involvement of the orbitofrontal cortex in the experience of regret. Science 304, 1167–1170 10.1126/science.1094550 15155951

[B9] Coricelli G. Dolan R. J. Sirigu A. (2007). Brain, emotion and decision making: the paradigmatic example of regret. Trends Cogn. Sci. 11, 258–265 10.1016/j.tics.2007.04.003 17475537

[B10] Deary I. J. Johnson W. Starr J. M. (2010). Are processing speed tasks biomarkers of cognitive aging? Psychol. Aging 25, 219–228 10.1037/a0017750 20230141

[B11] Eckert M. A. (2011). Slowing down: age-related neurobiological predictors of processing speed. Front. Neurosci. 5:25 10.3389/fnins.2011.0002 21441995 PMC3061488

[B12] Eisenberg N. (2005). The development of empathy-related responding, in Nebraska Symposium on Motivation (Lincoln, NE), 73 16342419

[B13] Elster J. (1999). Alchemies of the Mind. Cambridge, UK: Cambridge University Press

[B14] Epstude K. Roese N. J. (2008). The functional theory of counterfactual thinking. Pers. Soc. Psychol. Rev. 12, 168–192 10.1177/1088868308316091 18453477 PMC2408534

[B15] Fumagalli M. Ferrucci R. Mameli F. Marceglia S. Mrakic-Sposta S. Zago S. (2010a). Gender-related differences in moral judgments. Cogn. Process. 11, 219–226 10.1007/s10339-009-0335-2 19727878

[B16] Fumagalli M. Vergari M. Pasqualetti P. Marceglia S. Mameli F. Ferrucci R. (2010b). Brain switches utilitarian behavior: does gender make the difference? PLoS ONE 5:e8865 10.1371/journal.pone.0008865 20111608 PMC2810338

[B17] Gilbert D. T. Morewedge C. K. Risen J. L. Wilson T. D. (2004). Looking forward to looking backward the misprediction of regret. Psychol. Sci. 15, 346–350 10.1111/j.0956-7976.2004.00681.x 15102146

[B18] Gilligan C. (1982). In a Different Voice: Psychological Theory and Women's Development. Cambridge, MA: Harvard University Press

[B19] Girotto V. Ferrante D. Pighin S. Gonzalez M. (2007). Postdecisional counterfactual thinking by actors and readers. Psychol. Sci. 18, 510–515 10.1111/j.1467-9280.2007.01931.x 17576264

[B20] Girotto V. Legrenzi P. Rizzo A. (1991). Event controllability in counterfactual thinking. Acta Psychol. 78, 111–133 10.1016/0001-6918(91)90007-M21041524

[B21] Greene J. D. (2009). Dual-process morality and the personal/impersonal distinction: a reply to McGuire, Langdon, Coltheart, and Mackenzie. J. Exp. Soc. Psychol. 45, 581–584 10.1016/j.jesp.2009.01.003

[B22] Greene J. D. Nystrom L. E. Engell A. D. Darley J. M. Cohen J. D. (2004). The neural bases of cognitive conflict and control in moral judgment. Neuron 44, 389–400 10.1016/j.neuron.2004.09.027 15473975

[B23] Greene J. D. Sommerville R. B. Nystrom L. E. Darley J. M. Cohen J. D. (2001). An fMRI investigation of emotional engagement in moral judgment. Science 293, 2105–2108 10.1126/science.1062872 11557895

[B24] Harenski C. L. Antonenko O. Shane M. S. Kiehl K. A. (2008). Gender differences in neural mechanisms underlying moral sensitivity. Soc. Cogn. Affect. Neurosci. 3, 313–321 10.1093/scan/nsn026 19015084 PMC2607058

[B25] Johnson M. K. Sherman S. J. (1990). Constructing and reconstructing the past and the future in the present, Handbook of Motivation and Cognition: Foundations of Social Behavior, eds Higgins E. T. Sorrentino R. M. (New York, NY: The Guilford Press), 482–526

[B26] Kaasinen V. Någren K. Hietala J. Farde L. Rinne J. O. (2001). Sex differences in extrastriatal dopamine D2-like receptors in the human brain. Am. J. Psychiatry 158, 308–311 10.1176/appi.ajp.158.2.308 11156817

[B27] Kahneman D. Slovic P. Tversky A. (1982). Judgment Under Uncertainty: Heuristics and Biases. New York, NY: Cambridge University Press

[B28] Kimura D. (2002). Sex hormones influence human cognitive pattern. Neuro Endocrinol. Lett. 23, 67–77 12496737

[B29] Kohlberg L. (1964) Development of moral character and moral ideology in Review of Child Development Research, eds Hoffman M. L. Hoffman L. W. (New York, NY: Russell Sage Foundation), 383–432

[B35] Lebow R. N. (2007). Counterfactual thought experiments: a necessary teaching tool. Hist. Teacher 40, 153–176 10.2307/30036985

[B30] Mandel D. R. Hilton D. J. Catellani P. (2005). Psychology of Counter Factual Thinking. Oxford, UK: Routledge

[B31] McGuire J. Langdon R. Coltheart M. Mackenzie C. (2009). A reanalysis of the personal/impersonal distinction in moral psychology research. J. Exp. Soc. Psychol. 45, 577–580 10.1016/j.jesp.2009.01.002

[B32] Mikhail J. (2007). Universal moral grammar: theory, evidence and the future. Trends Cogn. Sci. 11, 143–152 10.1016/j.tics.2006.12.007 17329147

[B33] N'gbala A. Branscombe N. (1997). When does action elicit more regret than inaction and is counterfactual mutation the mediator of this effect? J. Exp. Soc. Psychol. 33, 324–343 10.1006/jesp.1996.1322

[B34] Nadelhoffer T. Feltz A. (2008). The Actor–Observer Bias and moral intuitions: adding fuel to sinnott-armstrong's fire. Neuroethics 1, 133–144 10.1007/s12152-008-9015-7

[B36] Rafetseder E. Cristi-Vargas R. Perner J. (2010). Counterfactual reasoning: developing a sense of “nearest possible world”. Child Dev. 81, 376–389 10.1111/j.1467-8624.2009.01401.x 20331674 PMC3201838

[B37] Rafetseder E. Schwitalla M. Perner J. (2013). Counterfactual reasoning: from childhood to adulthood. J. Exp. Child Psychol. 114, 389–404 10.1016/j.jecp.2012.10.010 23219156 PMC3582172

[B38] Riccardi P. Li R. Ansari M. S. Zald D. Park S. Dawant B. (2005). Amphetamine-induced displacement of &lsqb; 18f&rsqb; fallypride in striatum and extrastriatal regions in humans. Neuropsychopharmacology 31, 1016–1026 10.1038/sj.npp.1300916 16237395

[B39] Roese N. (2005). If Only: How to Turn Regret Into Opportunity. New York, NY: Random House Digital, Inc

[B40] Roese N. J. (1997). Counterfactual thinking. Psychol. Bull. 121, 133 10.1037/0033-2909.121.1.133 9000895

[B41] Roese N. J. Olson J. M. (2014). What Might Have Been: The Social Psychology of Counterfactual Thinking. New York, NY: Psychology Press

[B42] Schaich Borg J. Hynes C. Van Horn J. Grafton S. Sinnott-Armstrong W. (2006). Consequences, action, and intention as factors in moral judgments: an fMRIinvestigation. J. Cogn. Neurosci. 18, 803–817 10.1162/jocn.2006.18.5.803 16768379

[B43] Stalnaker R. (1968). Studies in Logical Theory. Oxford, UK: Blackweel

[B44] Thomson J. J. (1976). Killing, letting die, and the trolley problem. Monist 59, 204–217 10.5840/monist197659224 11662247

[B45] Woodward J. (2011). Psychological studies of causal and counterfactual reasoning in Understanding Counterfactuals, Understanding Causation, eds Hoerl C. McCormack T. Beck S. R. (Oxford, UK: Oxford University Press).

[B46] Young L. Koenigs M. (2007). Investigating emotion in moral cognition: a review of evidence from functional neuroimaging and neuropsychology. Br. Med. Bull. 84, 69–79 10.1093/bmb/ldm031 18029385

