# Supplementary material for: Counterfactual thinking in moral judgment: an experimental study
Source: Front Psychol. 2014 May 20;5:451. doi: 10.3389/fpsyg.2014.00451 (PMC4033199; doi:10.3389/fpsyg.2014.00451)
Supplement: Supplementary file 1 [file DataSheet1.DOCX]

APPENDIX A

Non moral dilemmas

1-Standard Turnips

You are a farm worker driving a turnip-harvesting machine. You are approaching two diverging paths. By choosing the path on the left you will harvest ten bushels of turnips. By choosing the path on the right you will harvest twenty bushels of turnips.

You have gathered ten bushels of turnips.

What could you have done to avoid this?

2-Plant Transport

Antonio is bringing home a number of plants from a store that is about two miles from his home. The trunk of his car, which he has lined with plastic to catch the mud from the plants, will hold most of the plants he has purchased. Antonio could bring all the plants home in one trip, but this would require putting some of the plants in the back seat as well as in the trunk. By putting some of the plants in the back seat he will ruin his fine leather upholstery which would cost thousands of dollars to replace.

The fine leather upholstery was ruined.

What John could have done to avoid this?

3-Scheduling

You are in charge of scheduling appointments in a dentist's office. Two people, Mr. Rossi and Mrs. Bianchi have called to make appointments for next Monday. The only available times for next Monday are at 10:00 and at 15:00. Mr. Rossi's schedule is rather flexible. He can have his appointment either at 10:00 or at 15:00. Mrs. Bianchi's schedule is less flexible. She can only have her appointment at 10.

You plan an appointment for Mrs. Bianchi at 15:00; she turns it down and calls another dentist.

What could you have done to avoid this?

4-Generic Brand

Elisa has a headache. She goes to the pharmacy with the intention of buying a particular name-brand headache medicine. Elisa discovers that the pharmacy is out of the brand you were looking for. The pharmacist, whom she has known for a long time and in whom she has a great deal of trust, tells her that he has in stock a generic product which is, in his words, "exactly the same" as the product Elisa had originally intended to buy.

Elisa still has a headache and has no drugs available.

What could Elisa have done to avoid this?

5-Cookies

You have decided to make a batch of cookies for yourself. You open your recipe book and find a recipe for cookies. The recipe calls for a cup of chopped walnuts. You don't like walnuts, but you do like macadamia nuts. As it happens, you have both kinds of nuts available to you.

When the cookies are ready, you decide to give them away because you do not like the flavor of the nuts.

What could you have done to avoid this?

6-Train or Bus

You need to travel from Milano to Padova in order to attend a meeting that starts at 14:00. You can take either the train or the bus. The train will get you there just in time for your meeting. The bus is scheduled to arrive an hour before your meeting, but the bus is occasionally several hours late because of traffic. It would be nice to have an extra hour before the meeting, but you cannot afford to be late.

You're late at the meeting.

What could you have done to avoid this?

7-Computer

Gabriele is planning to buy a new computer. At the moment the computer that he wants costs 1000 euro, but he has available only 700 euro. A friend who knows the computer industry has told him that this computer's price will drop to 500 euro next month. If Gabriele waits until next month to buy the new computer, he will have to use his old computer for a few weeks longer than he would like to. Nevertheless, Gabriele will be able to do everything he needs to do using his old computer during that time.

Gabriel asks for a loan to parents.

What could Gabriele have done to avoid this?

8- Survey

You're currently without a job and you're missing about 200 euro to pay the rent of your apartment. A representative of a reputable, national survey organization calls you at your home while you are having a quiet dinner by yourself. The representative explains that if you are willing to spend a half an hour answering questions about a variety of topics her organization will send you a check for 200 euro.

After a while you was thinking about how to retrieve the money.

What could you have done to avoid this?

9-Coupons

Sonia has gone to a bookstore to buy 50 euro worth of books. She has two coupons: one of these coupons gives 30% off of purchase price. This coupon expires tomorrow. The other coupon gives 25% off purchase price, and this coupon does not expire for another year.

The following week, Sonia goes back into same bookstore for a purchase without any coupon.

What could Sonia have done to avoid this?

10-Scenic Route

An old friend of Tommaso's has invited him to spend the weekend at his summer home, on the Amalfi Coast. Tommaso intends to travel there by car, and there are two routes: the highway and the coastal road. The highway will get him to his friend's house in about three hours, but the scenery along the highway is very boring. The coastal route will get him to his friend's house in about three hours and forty-five minutes, and the scenery along the coastal road is breathtakingly beautiful.

Tommaso arrives too late.

What Tommaso could have done to avoid this?

11- Reversed Turnips

Alessandro is a farm worker driving a turnip-harvesting machine. He is approaching two diverging paths.

By choosing the path on the left he will harvest thirty bushels of turnips. By choosing the path on the right he will harvest fifteen bushels of turnips.

Alexander has gathered fifteen bushels of turnips.

What could Alessandro have done to avoid this?

12-Investment Offer

You receive a letter from a reputable corporation that provides financial services. They invite you to invest in a mutual fund, beginning with an initial investment of 1000 euro.

You are familiar with this particular mutual fund. It has not performed very well over the past few years, and, based on what you know, there is no reason to think that it will perform any better in the future.

After a week the capital you invested in those actions has halved.

What could you have done to avoid this?

13. Broken VCR

Raffaele has brought your broken VCR to the local repair shop. The woman working at the shop tells him that the repair will cost about 100 euro. Raffaele noticed in the paper that morning that the electronics shop is having a sale on a VCR which is slightly better than old one for 120 euro.

After collecting the VCR repaired, the unit fails again.

What could Raffaele have done to avoid this?

14. Choosing Classes

Irene is beginning her senior year of college. In order to fulfill her graduation requirements, she needs to take a history class and a science class by the end of the year.

During the fall term, the history class is scheduled at the same time as the science class. During the spring term the same history class is offered, but the science class is not.

At the end of the year Irene cannot graduate.

What could Irene have done to avoid this?

15. Raffle

Lidia decided to buy a raffle ticket to support a local charity. They are separately raffling off two different cars: Car A and Car B. Lidia is a serious and knowledgeable car enthusiast, and she thinks that these two cars are equally good. Because there have been a lot of adds for Car B on TV recently, many more people have chosen to buy tickets for the Car B raffle. Since more people have bought tickets for the Car B raffle, her chances of winning are better in the Car A raffle than in the Car B raffle.

Lidia has not bought any lottery ticket.

What could Lidia have done to avoid this?

16. Jogging

You intend to accomplish two things this afternoon: going for a jog and doing some paperwork for tomorrow. In general you prefer to get your work done before you exercise. The weather is nice at the moment, but the weather forecast says that in a couple of hours it will start to rain.

At the end of the day you finish the project, but did not made any physical activity.

What could you have done to avoid this?

17. Food Prep

Giuseppe is preparing pasta with fresh vegetables, and he is deciding on the order in which he will do the various things he needs to do.. If he first starts the water boiling and then cuts the vegetables, he will be done in twenty minutes. If he cuts the vegetables and then starts the water boiling, he will be done in forty minutes.

When friends come, Giuseppe apologizes for the delay and asks them to wait.

What could Giuseppe have done to avoid this?

18. Shower

In your country there is water shortage because it has not rained for a long time. You are planning to attend a luncheon this afternoon, and before you go you will need to take a shower. You have some yard work that you would like to do before then, and doing this yard will cause you to perspire a fair amount. If you shower before you do your yard work. you will have to take another shower before the luncheon. At the moment, you're hot and you really want to take a cold shower.

You come to the dinner rumpled and smelly.

What could you have done to avoid this?

19. Errands

Today you have to do three errands: you need to go to the bakery in the morning and to the furniture store in the afternoon. You also need to go to the camera shop at some point. You prefer to do most of your errands in the morning, but you very much dislike doing unnecessary driving.

The camera shop is near the furniture store and far from the bakery. As a result you will have to drive less if you go to the camera shop in the afternoon when you go to the furniture store.

You decide to go to the camera shop in the morning and remain stuck in traffic.

What could you have done to avoid this?

20. New Job

You have been offered employment by two different firms, and you are trying to decide which offer to accept. Firm A has offered you an annual salary of 60.000 euro and fourteen days of vacation per year. Firm B has offered you an annual salary of 50,000 euro and sixteen days of vacation per year. The two firms and the two positions are otherwise very similar.

After a year of work you realize that your bank account is very low.

What could you have done to avoid this?

Moral impersonal dilemmas

21-Standard Trolley

Federico is at the wheel of a runaway trolley quickly approaching a bifurcation in the tracks. On the tracks extending to the left is a group of five railway workmen. On the tracks extending to the right is a single railway workman.

If Federico does nothing, the trolley will proceed to the left, causing the death of the five workmen. The only way to avoid the death of these workmen is to hit a switch on dashboard that will make the trolley proceed to the right, causing the death of the single workman.

Five workmen die.

What could Federico have done to avoid this?

22. Toxic Fumes

Giovanni is the late-night watchman in a hospital. Due to an accident in the next building, there are deadly fumes rising up through the hospital's ventilation system. In a certain room of the hospital there are three patients. In another room there is a single patient. If Giovanni does nothing the fumes will rise up into the room containing the three patients and cause their deaths.

The only way to avoid the deaths of these patients is to hit a switch: as a result of doing this the fumes will enter the room containing the single patient, causing his death.

After a few minutes three patients die.

What Federico could has done to avoid this?

23. Donation

You are at home one day when the mail arrives. You receive a letter from a reputable international aid organization. The letter asks you to make a donation of two hundred dollars to their organization.

The letter explains that a two hundred-dollar donation will allow this organization to provide needed medical attention to some poor people in another part of the world.

After a few days the news communicate the death of dozens of people for inadequate medical care.

What could you have done to avoid this?

24. Vaccine Policy

Laura works for the Bureau of Health. She is deciding whether or not your agency should encourage the use of a certain recently developed vaccine. 90% of people who take the vaccine develop an immunity to a deadly disease, but 10% of people who take the vaccine will actually get the disease that the vaccine is designed to prevent. All the available evidence, which is very strong, suggests that the chances of getting the disease due to lack of vaccination are much higher than the chances of getting the disease by taking the vaccine.

A very high number of people contract the disease.

What could Laura have done to avoid this?

25. Environmental Policy A1

You are a member of a government legislature. The legislature is deciding between two different policies concerning environmental hazards. Policy A has a 90% chance of causing no deaths at all and has a 10% chance of causing 1000 deaths. Policy B has a 92% chance of causing no deaths and an 8% chance of causing 10,000 deaths.

After one year, the deaths caused by the policy choice exceeds 9000.

What could you have done to avoid this?

26. Environmental Policy A2

Arturo is a member of a government legislature. The legislature is deciding between two different policies concerning environmental hazards.

Policy A has a 90% chance of causing no deaths at all and has a 10% chance of causing 1000 deaths. Policy B has an 88% chance of causing no deaths and a 12% chance of causing 10 deaths.

After one year, the deaths caused by the policy choice exceeds 1000.

What could Arturo have done to avoid this?

27. Sculpture

You are visiting the sculpture garden of a wealthy art collector. The garden overlooks a valley containing a set of train tracks. A railway workman is working on the tracks, and an empty runaway trolley is heading down the tracks toward the workman. The only way to save the workman's life is to push one of the art collector's prized sculptures down into the valley so that it will roll onto the tracks and block the trolley's passage.

After a few minutes the workman dies.

What could you have done to avoid this?

28. Speedboat

Simone is on vacation on a remote island, and he's fishing from a seaside dock. He observes a group of tourists board a small boat and set sail for a nearby island. Soon after their departure he hears on the radio that there is a violent storm brewing, a storm that is sure to intercept them.

The only way that Simone has to ensure their safety is to warn them by borrowing a nearby speedboat. The speedboat belongs to a miserly tycoon who will not be happy about this.

At the end of the day Simone hears on the radio that the tourists group is missing.

What could Simone have done to avoid this?

29. Guarded Speedboat

While on vacation on a remote island, you are fishing from a seaside dock. You observe a group of tourists board a small boat and set sail for a nearby island. Soon after their departure you hear over the radio that there is a violent storm brewing, a storm that is sure to intercept them.

The only way that you have to ensure their safety is to warn them by borrowing a nearby speedboat. The speedboat belongs to a miserly tycoon who has hired a fiercely loyal guard to make sure that no one uses his boat without permission. To get to the speedboat you will have to lie to the guard.

At the end of the day you hear on the radio that the tourists group is missing.

What could you have done to avoid this?

30. Five-for-Seven Trolley

You are at the wheel of a runaway trolley quickly approaching a bifurcation in the tracks. On the tracks extending to the left there is a group of seven railway workmen. On the tracks extending to the right there is a group of five railway workmen.

If you do nothing the trolley will proceed to the left, causing the death of the seven workmen. The only way to save these workmen is to hit a switch on your dashboard that will cause the trolley to proceed to the right, causing the death of the five workmen on the other side.

Seven workmen die.

What could you have done to avoid this?

31. Three-for-Seven Fumes

You are the late-night watchman in a hospital. Due to an accident in the building next door, there are deadly fumes rising up through the hospital's ventilation system. In a room of the hospital there are three patients. In another room there are seven patients. If you do nothing the fumes will rise up into the room containing the seven patients and cause their death. The only way to save these patients is to hit a switch, which will cause the fumes to bypass the room containing the seven people. As a result of doing this. the fumes will enter the room containing the three patients, causing their death.

After a few minutes seven patients die.

What could you have done to avoid this?

32. Resume

Giulio has been trying to find a job lately without much success. He figured that he would be more likely to get hired if he had a more impressive resume.

He decided to put some false information on his resume in order to make it more impressive. By doing this he ultimately managed to get hired, beating out several candidates who were actually more qualified than he is.

After a few days he is discovered and immediately fired.

What could Giulio have done to avoid this?

33. Taxes

You are the owner of a small business trying to make ends meet. It occurs to you that you could lower your taxes by pretending that some of your personal expenses are business expenses.

For example, you could pretend that the stereo in your bedroom is being used in the lounge at the office, or that your dinners out with your wife are dinners with clients.

After a few months you close the business and 10 peoples lose their jobs.

What could you have done to avoid this?

34. Stock Tip

Alessandro is a management consultant working for a large corporate client. He has access to confidential information that would be very useful to investors. He has a friend who gambles on the stock market. He owes this friend a sizable sum of money. By providing her with certain confidential information, he could help her make a lot of money, considerably more than what he owes her. Releasing information in this way is strictly forbidden by federal law.

Alessandro still has to pay her friend.

What could Alessandro have done to avoid this?

35. Illegal Lunch

You are a lawyer working on a big case. The judge presiding over the trial happens to be someone you know from law school. The two of you were rather friendly back then, but now, decades later, it seems that your old friend barely remembers you. You're quite sure that if you were to talk to him over lunch, you could help his memory and he would begin to see you as an old buddy, which would be very good for your work on this case. It is illegal for judges and lawyers working on the same case to meet socially.

You lose the case.

What could you have done to avoid this?

36. Environmental Policy B1

Marco is a member of a government legislature. The legislature is deciding between two different policies concerning environmental hazards.

Policy A has a 90% chance of causing no deaths at all and has a 10% chance of causing 1000 deaths. Policy B has a 92% chance of causing no deaths and an 8% chance of causing 10,000 deaths.

After one year, the deaths caused by the policy choice exceed 9000.

What could Marco have done to avoid this?

37. Environmental Policy B2

You are a member of a government legislature. The legislature is deciding between two different policies concerning environmental hazards.

Policy A has a 90% chance of causing no deaths at all and has a 10% chance of causing 1000 deaths. Policy B has a 88% chance of causing no deaths and a 12% chance of causing 10 deaths.

After one year, the deaths caused by the policy choice exceeds 1000.

What could you have done to avoid this?

38. Lost Wallet

You are walking down the street when you come across a wallet lying on the ground. You open the wallet and find that it contains several hundred euro in cash as well the owner's driver's license. From the credit cards and other items in the wallet it's very clear that the wallet's owner is wealthy. You, on the other hand, have been hit by hard times recently and could really use some extra money.

You don’t know how to pay electricity and gas.

What could you have done to avoid this?

39. Eyes

Daniele is a veteran of a war during which both his eyes were amaged. Due to recent medical advances, it is now possible to perform eye transplants, but there are no willing donors. A black-market surgeon offers to help him. If he hires him, he and his associates will kidnap a randomly selected stranger, carve out one of his eyes, and transfer it to Daniele.

Daniel remains blind.

What could Daniele have done to avoid this?

40. University exam

Luigi attends college. He attends the final year of Engineering; he misses only an exam before graduation. This last exam is very difficult. One day, walking through the halls, he finds the professor's office door open. On the desk there is the test that the professor prepared for the exam. Having it means getting a high grade and graduation assured.

Luigi does not pass the test. He will graduate in six months.

What could Luigi have done to avoid this?

Moral personal dilemmas

41-Transplant

You are a doctor. You have five patients, each of whom is about to die due to a failing organ. You have another patient who is healthy.

The only way that you have to save the lives of the first five patients is to transplant five of this young man's organs (against his will) into the bodies of the other five patients. If you do this, the young man will die, but the other five patients will live.

Few days later five patients die.

What could you have done to avoid this?

42. Footbridge

A runaway trolley is heading down the tracks toward five workmen who will be killed if the trolley proceeds on its present course. You are on a footbridge over the tracks, in between the approaching trolley and the five workmen. Next to you on this footbridge is a stranger who happens to be very large.

The only way to save the lives of the five workmen is to push this stranger off the bridge and onto the tracks below, where his large body will stop the trolley.

Five workmen die.

What could you have done to avoid this?

43. Country Road

Arturo is driving along a country road when he hears a call for help coming from some roadside bushes. He pulls over and encounters a man whose legs are covered with blood. The man explains that he has had an accident while hiking and asks him to take him to a nearby hospital.

His initial inclination is to help this man, who will probably lose his leg if he does not get to the hospital soon. However, if Arturo gives this man a lift, the blood will ruin the leather upholstery of her car.

After a few hours the man's leg is amputated.

What could Luigi have done to avoid this?

4. Architect

Ilenia is a young architect visiting one construction site with her boss. The boss is a despicable individual who makes everyone around him miserable, including her.

It occurs to her that if she were to push him off of the building that they are inspecting, he would fall to his death and everyone would think this was an accident.

Ilenia is very unhappy because of the boss’ oppression.

What could Ilenia have done to avoid this?

45. Hard Times

You are the father of two sons and one daugther; you are a poor household in a developing country. Your crops have failed for the second year in a row, and it appears that you have no way to feed your family.

You know a man from your village who lives in the city and who makes sexually explicit films featuring small children such as your daughter. He tells you that in one year of working in his studio your daughter could earn enough money to keep your family fed for several growing seasons.

Your children die from undernutrition.

What could you have done to avoid this?

7. Smother for dollars

Sebastiano is in hospital lounge waiting to visit his sick mother that needs very expensive medical care that Sebastiano cannot pay. A young man sitting next to him explains that his father is very ill. The doctors believe that he has a week to live at most. He explains further that his father has a substantial life insurance policy that expires at midnight. If his father dies before midnight, this young man will receive a very large sum of money. He offers to Sebastiano half a million euros to go up to his father's room and smother his father with a pillow.

Sebastiano's mother dies after a few months.

What could Sebastiano have done to avoid this?

47. Crying Baby

Enemy soldiers have taken over your village. They have orders to kill all remaining civilians. You and some of your townspeople have sought refuge in the cellar of a large house. Outside you hear the voices of soldiers who have come to search the house for valuables.

Your baby begins to cry loudly. You cover his mouth to block the sound. If you remove your hand from his mouth his crying will summon the attention of the soldiers who will kill you, your child, and the others hiding out in the cellar. To save yourself and the others you must smother your child to death.

You are discovered and killed.

What could you have done to avoid this?

48. Plane Crash

Your plane has crashed in the Himalayas. The only survivors are yourself, another man, and a young boy. Your only chance at survival is to find your way to small a village on the other side of the mountain, several days away.

The boy has a broken leg and cannot move very quickly. His chances of surviving the journey are essentially zero. Without food, you and the other man will probably die as well. The other man suggests that you sacrifice the boy and eat his remains over the next few days.

You all die of starvation.

What could you have done to avoid this?

49. Hired Rapist

Michele has been dissatisfied with his marriage for several years. It is his distinct impression that his wife no longer appreciates him. He remembers how she appreciated him years ago when he took care of her after she was mugged. Michele devises the following plan to regain her wife's affection.

He will hire a man to break into house while he is away. This man will tie up his wife and rape her. He, upon hearing the horrible news, will return swiftly to her side, to take care of her and comfort her, and she will once again appreciate him.

Michele’s marriage is always in a crisis.

What could Michele have done to avoid this?

50. Sophie's Choice

It is wartime and Sofia and her two children, aged eight and five, are living in a territory that has been occupied by the enemy. At the enemy's headquarters there is a doctor who performs painful experiments on humans that inevitably lead to death.

He intends to perform experiments on one of her children, but he will allow her to choose which of her children will be experimented upon. Sofia has twenty-four hours to bring one of her children to his laboratory. If she refuses to bring one of her children to his laboratory, he will experiment on both of them.

The next day both Sofia’s children die.

What could Sofia have done to avoid this?

51. Infanticide

Monia is a fifteen-year-old girl who has become pregnant. By wearing loose clothing and deliberately putting on weight she has managed to keep her pregnancy a secret. One day, while at school, there is a breaking of the waters. She runs to the girls locker room and hides for several hours while she delivers the baby. Monia is sure that she is not prepared to care for this baby.

She thinks that it would be such a relief to simply clean up the mess she has made in the locker room, wrap the baby in some towels, throw the baby in the dumpster behind the school, and act as if nothing had happened.

After a few days Monia reads in the newspaper of the death of a child found in a dumpster near the school.

What could Monia have done to avoid this?

52. Lifeboat

You are on a cruise ship when there is a fire on board, and the ship has to be abandoned. The lifeboats are carrying many more people than they were designed to carry. The seas start to get rough, and the boat begins to fill with water. If nothing is done it will sink before the rescue boats arrive and everyone on board will die. However, there is an injured person who will not survive in any case. If you throw that person overboard the boat will stay afloat and the remaining passengers will be saved.

Everybody drowns..

What could you have done to avoid this?

53. Preventing the Spread

You are a waiter. You overhear one of your customers say that he is about to go to jail and that in his last forty-eight hours of freedom he plans to infect as many people as possible with HIV. You know him well enough to know that he is telling the truth and that he has access to many potential victims.

You happen to know that he has a very strong allergy to poppy seeds. If he eats even only one he will have a seizure and have to be hospitalized. for at least forty-eight hours.

A few days later, you read in the newspaper of several rapes in the city.

What could you have done to avoid this?

54. Safari

Luisa is part of a group of ecologists who live in a remote stretch of jungle. The entire group, which includes eight children, has been taken hostage by a group of paramilitary terrorists. One of the terrorists takes a liking to her. He informs Luisa that his leader intends to kill her and the rest of the hostages the following morning. He is willing to help her and the children escape, but as an act of good faith, he wants that she kills one of fellow hostages whom he does not like.

Next morning the leader of paramilitary group kills the whole group.

What could Luisa have done to avoid this?

55. Bomb

You are negotiating with a powerful and determined terrorist who is about to set off a bomb in a crowded area. Your one advantage is that you have his teen-age son in your custody. There is only one thing that you can do to stop him from detonating his bomb, which will kill thousands of people if detonated. To stop him, you must contact him over the satellite hook-up that he has established and, in front of the camera, break one of his son's arms and then threaten to break the other one if he does not give himself up.

After a few minutes lots of people die and are injured.

What could you have done to avoid this?

56. Submarine

Massimiliano is the captain of a military submarine travelling underneath a large iceberg. An onboard explosion has caused Massimiliano to lose most of his oxygen supply and has injured one of the crew who is quickly losing blood. The injured crew member is going to die from his wounds no matter what happens.

The remaining oxygen is not sufficient for the entire crew to make it to the surface. The only way to save the other crew members is to shoot dead the injured crew member so that there will be just enough oxygen for the rest of the crew to survive.

Everyone dies before reaching the surface.

What could Massimiliano have done to avoid this?

57. Lawrence of Arabia

You are the leader of a small army that consists of warriors from two tribes, the hill tribe and the river tribe. You belong to neither tribe. During the night a hill tribesman got into an argument with a river tribesman and murdered him. The river tribe will attack the hill tribe unless the murderer is put to death, but the hill tribe refuses to kill one of its own warriors.

The only way for you to avoid a war between the two tribes that will costs hundreds of lives is to publicly execute the murderer by cutting off his head with your sword.

A bloody war begins that brings the two tribes to extermination.

What could you have done to avoid this?

58. Sacrifice

Stefania, her husband and their four children are crossing a mountain range on the return journey to her homeland. She has inadvertently set up camp on a local clan's sacred burial ground.

The leader of the clan says that according to the local laws, her and family must be put to death. However, he will let Stefania her husband and three other children alive if she herself will kill her oldest son.

After a few minutes are all killed.

What could Stefania have done to avoid this?

59. Vaccine Test

A viral epidemic has spread across the globe killing millions of people. You have developed two drugs in your home laboratory. You know that one of them is a vaccine, but you don't know which one. You also know that the other one is deadly. You have with you two people who are under your care.

Millions of people die around the world.

What could you have done to avoid this?

60. Euthanasia

You are the leader of a small group of soldiers. You are on your way back from a completed mission in enemy territory when one of your men has stepped in trap that has been set by the enemy and is badly injured. The trap is connected to a radio device that by now has alerted the enemy of your presence. They will soon be on their way.

If the enemy finds your injured man they will torture him and kill him. He begs you not to leave him behind, but if you try to take him with you your entire group will be captured.

The enemy reaches you and kills the whole group including yourself.

What could you have done to avoid this?
